# Direct-acting antiviral treatment for Hepatitis C Virus in geriatric patients: a real-world retrospective comparison between early and late elderly patients

**DOI:** 10.7717/peerj.10944

**Published:** 2021-03-16

**Authors:** Hsu-Heng Yen, Pei-Yuan Su, I-Ling Liu, Ya-Yuei Zeng, Siou-Ping Huang, Yu-Chun Hsu, Chia-Wei Yang, Yang-Yuan Chen

**Affiliations:** 1 Division of Gastroenterology, Department of Internal Medicine, Changhua Christian Hospital, Changhua, Taiwan; 2 General Educational Center, Chienkuo Technology University, Changhua, Taiwan; 3 Institute of Medicine, Chung Shan Medical and Dental College, Taichung, Taiwan

**Keywords:** Hepatitis C, Geriatric, Viral hepatitis, Direct-acting antiviral treatment

## Abstract

**Introduction:**

Chronic hepatitis C virus (HCV) infection rates are high in the geriatric population considering that interferon-based therapy is usually intolerable. With the introduction of oral antiviral therapy for HCV, increased treatment tolerability and good treatment responses have been observed. However, treatment data regarding the geriatric population have been limited. Therefore, this retrospective study aimed to evaluate the efficacy and safety of direct-acting antiviral therapy for HCV in the geriatric population.

**Materials and Methods:**

The primary end point was sustained virologic response (SVR) 12 weeks after treatment completion, whereas the secondary end points were treatment-related side effects and short-term survival rate following treatment.

**Results:**

In total, 492 patients (median age, 73 years; 43.9% males), including 278 early elderly patients, were enrolled. Among the included patients, 45% had cirrhosis. HCV genotypes 1 (72.4%) and 2 (25.4%) were the most common. The overall SVR rate was 96.7%, with no difference in SVR rates observed between early and late elderly groups (96.8% vs. 96.7%; *p* = 0.983). Both groups showed similar side effects, including dizziness (11.4%), and fatigue (8.7%), with three patients discontinuing therapy owing to side effects. Both groups had a similar 3-year survival rate. Significant factors associated with post-treatment survival included cirrhosis, albumin, and creatinine level.

**Conclusions:**

Our real-world data showed that both early and late elderly patients could undergo direct-acting antiviral treatment for HCV with excellent treatment outcomes.

## Introduction

Hepatitis C virus (HCV) infection has been associated with significant liver disease-related morbidity and mortality (Liu et al., 2020a). Moreover, recent studies have demonstrated that HCV infection was associated with extrahepatic complications, such as increased rate of lymphoproliferative disorder, rheumatic disorders, and extrahepatic malignancy (Huang et al., 2020b; Lee et al., 2019; Polo & Laufer, 2017; Soriano et al., 2016). Persistent systemic inflammation can also promote increased risk for renal, neurologic, and cardiovascular disease progression (Huang et al., 2020a; Wu et al., 2018b; Yen et al., 2012; Yen et al., 2020). Thus, control of HCV infection may help prevent extrahepatic complications despite mild liver involvement. Interferon-based (IFN) therapy has been the gold standard treatment for HCV infection since the early 21st century. Although 60%–80% of patients receiving IFN-based therapy are ultimately cured of HCV infection, significant side effects from such a therapy have limited its widespread use among vulnerable populations, such as those with HIV infection, renal insufficiency, and older age (Ansaldi et al., 2014; Liu et al., 2013; Liu et al., 2020b; Yen et al., 2020). Increased rates of HCV infection and HCV-related liver fibrosis have been reported in the geriatric population (Huang & Yu, 2017). However, previous studies have shown that IFN-based therapy had reduced therapeutic efficacy among HCV-infected geriatric patients (Hu et al., 2013; Huang & Yu, 2017; Roeder et al., 2014), with frequent dose reductions or treatment discontinuations. Thus, IFN-based HCV therapy had only been considered for geriatric patients who had significant hepatic fibrosis and no other health problems (Cainelli, 2008; Huang & Yu, 2017).

The recent introduction of direct-acting antiviral (DAA) therapy has revolutionized HCV treatment with its high sustained virologic response (SVR) rates (>90%) and good safety profile. However, geriatric patients are frequently excluded from clinical trials owing to the presence of concurrent comorbidities. Furthermore, data regarding treatment outcomes among the late elderly patients aged >75 years have been considerably lacking (Huynh & Hu, 2020; Villani et al., 2019). Costs for these new agents have been reimbursed by the Taiwanese health care system since 2017, with the aim of achieving 80% DAA treatment coverage rate by 2025 (Burki, 2019; Chen et al., 2019). Although most patients infected with HCV can currently be treated with oral DAAs, real-world data on the safety and effectiveness of these agents among different geriatric population remain limited. Thus, the current study aimed to present our real-world experience with anti-HCV therapy in the geriatric population.

## Materials & Methods

### Materials

This retrospective study enrolled patients who fulfilled the following criteria for analysis: patients referred for hepatitis C therapy with HCV viremia, age of ≥65 years; and received ≥1 dose of anti-HCV therapy between January 2017 and December 2019 at the Changhua Christian Hospital. Patients who ever received DAA therapy or generic were excluded. Under the approval by Changhua Christian Hospital Institutional Review Board (CCH IRB No 200403 and 190814) with the consideration of the retrospective design of the study, informed consent was waived. Medical information was extracted from the electronic medical records regarding the comorbid conditions, liver cirrhosis status, anti-HCV treatment regimen and duration, laboratory values, and adverse events. All procedures were performed under Changhua Christian Hospital guidelines and regulations.

### Evaluation of treatment, efficacy, and safety

The current study aim is to compare treatment responses between early elderly (aged 65–74 years) and late elderly (aged >75 years) patients (Hori et al., 2014). ART HCV assays (RealTime HCV and HCV Genotype II, Abbott Molecular, Abbott Park, IL, USA) were used for quantifying HCV RNA concentrations and genotyping. After completing the treatment course, end-of-treatment viral response (ETVR) was defined as HCV RNA level below the lower limit of quantification (LLOQ). SVR was defined as an HCV RNA level below the LLOQ 3 months after the last dose. Data were collected as our previously described work (Liu et al., 2020a; Liu et al., 2020b; Yen et al., 2020). Specifically The treatment period ranged from 8 to 24 weeks according to the medication package insert considering cirrhosis status and HCV genotype. The selected DAA regimen depended on patient preference after evaluating potential drug interactions and risks and benefits of therapy as well as discussions with the attending physician. Liver cirrhosis was diagnosed based on liver biopsy, ultrasound, or the endoscopic evidence of varices. SVR was divided into two groups: intention-to-treat group (ITT), which includes patients receiving at least one dose of DAA, and per-protocol group (PP), excluding patients owing to non-virological failure. Premature treatment discontinuation rate was also analyzed.

### Statistical analyses

Data are expressed as n (%) or n/N(%), median (interquartile range) as the distribution of continuous variables was non-normal as per one-sample Kolmogorov–Smirnov test. The data of normal distribution are expressed as median and standard deviation. The continuous variables of normal distribution could be compared using *t*-test. Categorical variables were compared using the chi-square test with Yate’s correction or the Fisher’s exact test; continuous variables were compared using the Mann–Whitney *U*-test as appropriate. The association between clinical factors and post-treatment survival was evaluated using univariate and multivariate analyses. The Cox proportional hazards model was adjusted for confounding clinical variables (age, male, cirrhosis, cancer, DM, hypertension, and ascites), including, by default, the backward elimination procedure used for the potential variables (FIB4, HCV RNA, comorbidity number, interferon therapy, BMI, creatinine, eGFR, ALT, INR, bilirubin, albumin, and hemoglobin) in the multivariable model. Risk was expressed as hazard ratios (HRs) and 95% confidence intervals (CIs). All statistical analyses were performed using SPSS version 18.0 and Medcalc version 19.3, with *p* values of <0.05 indicating statistical significance.

## Results

### Comparison of baseline variables between early and late elderly patients

**Figure 1:**
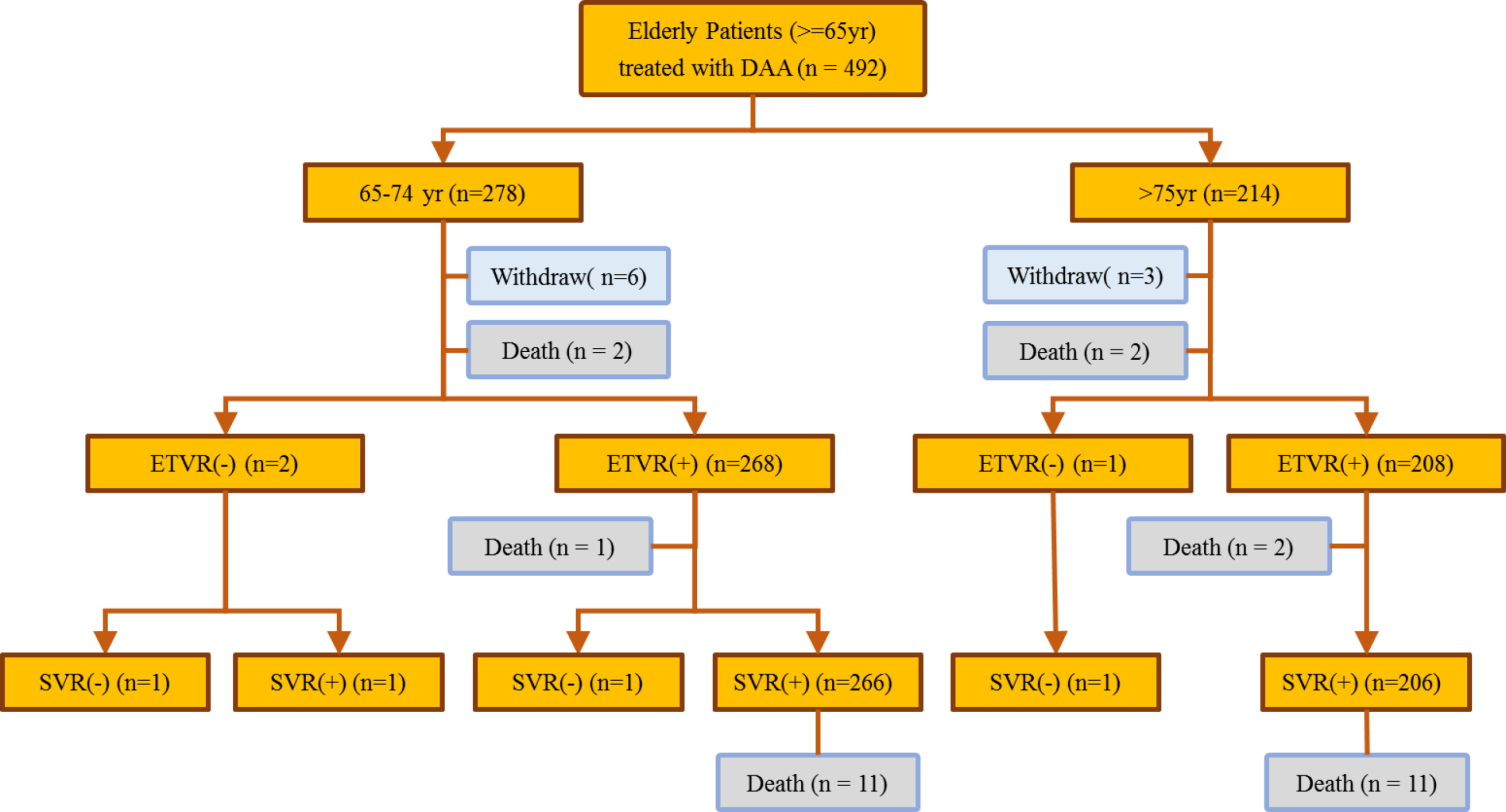
Flowchart showing patient outcome.

A total of 492 patients with HCV infection (predominantly female, 56.1%; median age, 73 years) received anti-HCV therapy during the study period (Fig. 1). Among the included patients, 56.5% belonged to the early elderly group, whereas 224 (45.5%) had liver cirrhosis. The most common HCV genotype observed was type 1 (72.4%), followed by type 2 (25.4%) and others (2.2%). Hepatitis B co-infection was noted in 5% of the patients. The late elderly group had greater serum creatine levels, FIB-4 scores, genotype 1 infections, and interferon-naïve patients than the early elderly group. The late elderly group had lower glutamic oxaloacetic transaminase (GOT), bilirubin, albumin, hemoglobin levels, and body mass index than the early elderly group. Among the included patients, 14.8% had malignancies, whereas 7.9% had liver-related malignancies. Comorbidity profiles were similar between both groups. Four fatalities were observed during the treatment period, all of which were determined to be unrelated to therapy, whereas 22 fatalities were observed after the treatment. Patient characteristics are summarized in Table 1 and Fig. 1.

**Table 1:** Descriptive characteristics of study participants.

**Characteristics**	**All patients (*N* = 492)**	**Age 65–74 years (*N* = 278)**	**Age ≥ 75 (*N* = 214)**	**PValue**
Age, years, median (IQR)	73 (68–78)	69 (67–71)	79 (77–83)	<0.001
Gender-male, n (%)	216 (43.9)	128 (46)	88 (41.1)	0.318
BMI, kg/m^2^, median (IQR)	23.565 (21.33–26.015)	23.81 (21.76–26.55)	23.155 (21.08–25.4)	0.004
CRE, mg/dL, median (IQR)	0.92 (0.68–1.17)	0.84 (0.66–1.12)	0.97 (0.8–1.26)	<0.001
eGFR, mL/min/1.73 m^2^, median (IQR)	72.915 (54.78–89.98)	78.79 (60.79–93.97)	63.535 (47.08–84.22)	<0.001
ALT, U/L, median (IQR)	47.5 (29.5–81)	57 (30–91)	42.5 (29–66)	0.002
INR, median (IQR)	0.97 (0.92–1.03)	0.97 (0.92–1.03)	0.97 (0.91–1.04)	0.794
BIL, mg/dL, median (IQR)	0.74 (0.58–1.01)	0.8 (0.6–1.04)	0.7 (0.56–0.97)	0.019
ALB, g/dL, median (IQR)	3.8 (3.5–4.1)	3.9 (3.6–4.2)	3.7 (3.5–4)	<0.001
HB, g/dL, median (IQR)	13 (11.5–14.2)	13.4 (12.1–14.4)	12.55 (11.2–13.6)	<0.001
FIB-4, median (IQR)	3.885 (2.655–6.025)	3.51 (2.38–5.72)	4.355 (3.31–6.28)	<0.001
Cirrhosis, n (%)	224 (45.5)	119 (42.8)	105 (49.1)	0.197
Hepatitis B, n (%)	27 (5.5)	15 (5.4)	12 (5.6)	1.000
Hepatitis B DNA, IU/mL, median (IQR)^a^	147.5 (61–208)	112 (50–143)	193 (152–208)	0.175
**Comorbidity, n (%)**				
CHD	1 (0.2)	1 (0.4)	0	1.000
HIV	6 (1.2)	5 (1.8)	1 (0.5)	0.240
DM	93 (18.9)	48 (17.3)	45 (21)	0.347
HTN	151 (30.7)	77 (27.7)	74 (34.6)	0.123
CVA	13 (2.6)	5 (1.8)	8 (3.7)	0.295
Any Cancer	73 (14.8)	43 (15.5)	30 (14)	0.749
Liver Cancer	39 (7.9)	22 (7.9)	17 (7.9)	1.000
HCV RNA, log10 IU/mL, median (IQR)	5.77 (4.81–6.27)	5.8 (4.82–6.28)	5.74 (4.79–6.26)	0.649
HCV genotype, n (%)				0.038
Type 1^b^	356 (72.4)	188 (67.6)	168 (78.5)	
Type 2^c^	125 (25.4)	81 (29.1)	44 (20.6)	
Type 4	3 (0.6)	3 (1.1)	0	
Type 6	5 (1)	3 (1.1)	2 (0.9)	
Mix Type^d^	3 (0.6)	3 (1.1)	0	
Treatment week, n (%)				0.411
8 weeks	64 (13)	41 (14.7)	23 (10.7)	
12 weeks	395 (80.3)	218 (78.4)	177 (82.7)	
24 weeks	33 (6.7)	19 (6.8)	14 (6.5)	
DAA, n (%)				
Sofosbuvir/ledipasvir	224 (45.5)	107 (38.5)	117 (54.7)	<0.001
Glecaprevir/pibrentasvir	90 (18.3)	56 (20.1)	34 (15.9)	0.274
Ombitasvir/paritaprevir/ritonavir/dasabuvir	74 (15)	54 (19.4)	20 (9.3)	0.003
Others^e^	104(21.1)	61(21.9)	43(20.1)	0.699
Prior interferon therapy, n (%)				0.022
Interferon failure	72 (14.6)	51 (18.3)	21 (9.8)	
Interferon naive	411 (83.5)	221 (79.5)	190 (88.8)	
Interferon interruption	9 (1.8)	6 (2.2)	3 (1.4)	
Child–Pugh Classification for cirrhotic patients, n/N(%)				0.726
A	216/224 (96.4)	114/119 (95.8)	102/105 (97.1)	
B	8/224 (3.6)	5/119 (4.2)	3/105(2.9)	
Ascites, n (%)				0.461
None	476 (96.7)	268 (96.4)	208 (97.2)	
Mild	14 (2.8)	8 (2.9)	6 (2.8)	
Moderate	2 (0.4)	2 (0.7)	0	

**Notes.**

BMI body mass index ALT alanine aminotransferase BIL bilirubin ALB albumin CRE Creatinine INR international normalized ratio HB haemoglobin FIB-4 Fibrosis-4 CHD coronary heart disease HIV Human immunodeficiency virus DM diabetes mellitus HTN hypertension CVA cerebrovascular accident HCV hepatitis C virus DAA direct antiviral agent eGFR estimated Glomerular filtration rate

a HBV DNA data was available for only 10 patients

b HCV genotype 1 including 1, 1a, 1b

c HCV genotype 2 including 2, 2a, 2b

d Mix type including 1or 6, 3, or 4

e Other DAA regimen, including Daclatasvir/asunaprevir, Sofosbuvir/ribavirin, Sofosbuvir/daclatasvir, Elbasvir/grazoprevir, Sofosbuvir/velpatasvir

### Comparison of treatment regimens and virologic responses

Sofosbuvir and ledipasvir were the most prescribed DAAs (45.5%), followed by glecaprevir and pibrentasvir (18.3%), ombitasvir/paritaprevir/ritonavir/dasabuvir (15%), and other DAAs (21.1%). Late elderly patients received more sofosbuvir/ledipasvir-based therapy than early elderly patients (54.7% vs. 38.5%; *p* < 0.001). More than 91% of the patients were able to complete the treatment course, with no difference in treatment duration or regimen having been observed between both groups. A total of 3 patients had a virologic treatment failure and 13 patients had non-virological treatment failure. The treatment was prematurely terminated in 13 patients, including deaths (*n* = 7), loss to follow-up (*n* = 1), discontinuation owing to patient request (*n* = 2), discontinuation owing to side effect (*n* = 2), and liver decompensation (*n* = 1). The overall ITT ETVR and PP SVR rate was 97.0% and 99.4%, whereas the overall ITT SVR and PP SVR rate was 96.7% and 99.4%, respectively. Both groups had similar treatment virologic responses (Table 2).

**Table 2:** End of treatment and sustained virological responses.

**HCV RNA <LLOQ**^a^	**All patients (*N* = 492)**		**Age 65–74 years (*N* = 278)**		**Age ≥ 75 (*N* = 214)**		**P Value**
	n/N (%)	95% CI	n/N (%)	95% CI	n/N (%)	95% CI	
**End of Treatment Response (ETVR)**							
Per Protocol Analysis	476 / 479 (99.4)	98.2–99.9	268 / 270 (99.3)	97.4–99.9	208 / 209 (99.5)	97.3–100	1.000
Intention to Treat	477 / 492 (97.0)	95.1–98.3	269 / 278 (96.8)	94.0–98.5	208 / 214 (97.2)	94–99	0.990
**Sustained Response (SVR)**							
Per Protocol Analysis	473 / 476 (99.4)	98.2–99.9	267 / 269 (99.3)	97.4–99.9	206 / 207 (99.5)	97.3–100	1.000
Intention to Treat	476 / 492 (96.7)	94.7–98.1	269 / 278 (96.8)	94.0–98.5	207 / 214 (96.7)	93.3–98.7	1.000

**Notes.**

a LLOQ, lower limit of qualification is 12 IU/mL

### Safety profile comparison

Pruritus was the most reported side effect of treatment, followed by dizziness and fatigue (Table 3). Grade 2 and 3 anemia was observed in 11.6% and 3.1% of the patients, respectively, whereas <2.7% exhibited a significant increase in bilirubin, GOT, and GPT levels. Among the 26 HBV and HCV co-infected patients, one patient had elevated HBV DNA level requiring anti-HBV therapy during anti-HCV therapy. There is no HBV reactivation-related complication in the study population.

**Table 3:** Safety summary of the study participant.

**Side effects, n (%)**	**All patients (*N* = 492)**	**Age 65**–**74 years (*N* = 278)**	**Age ≥ 75 (*N* = 214)**	**P value**
Fatigue	43 (8.7)	27 (9.7)	16 (7.5)	0.478
Nausea	13 (2.6)	7 (2.5)	6 (2.8)	1.000
Pruritus	66 (13.4)	46 (16.5)	20 (9.3)	0.029
Dizziness	56 (11.4)	30 (10.8)	26 (12.1)	0.744
**Laboratory adverse event**	**All patients (*N* = 490)**	**Age 65**–**74 years (*N* = 276)**^a^	**Age ≥ 75 (*N* = 214)**	**P value**
**Anemia**^b^				0.041
G1	418 (85.3)	244 (88.4)	174 (81.3)	
G2	57 (11.6)	23 (8.3)	34 (15.9)	
G3	15 (3.1)	9 (3.3)	6 (2.8)	
**Bilirubin**				0.535
1.5–3 × elevation	38 (7.8)	23 (8.3)	15 (7)	
≥3 × elevation	13 (2.7)	9 (3.3)	4 (1.9)	
**AST**				0.444
3–5 × elevation	12 (2.4)	5 (1.8)	7 (3.3)	
≥5 × elevation	4 (0.8)	3 (1.1)	1 (0.5)	
**ALT**				0.439
3–5 × elevation	12 (2.4)	5 (1.8)	7 (3.3)	
≥5 × elevation	5 (1)	2 (0.7)	3 (1.4)	

**Notes.**

a Two patients who withdrew before ETVR were not included for analysis of adverse events.

b The anemia grade was in accordance with the Common Terminology Criteria for Adverse Events version 5.0.

### Short-term survival analysis post anti-HCV therapy

**Figure 2:**
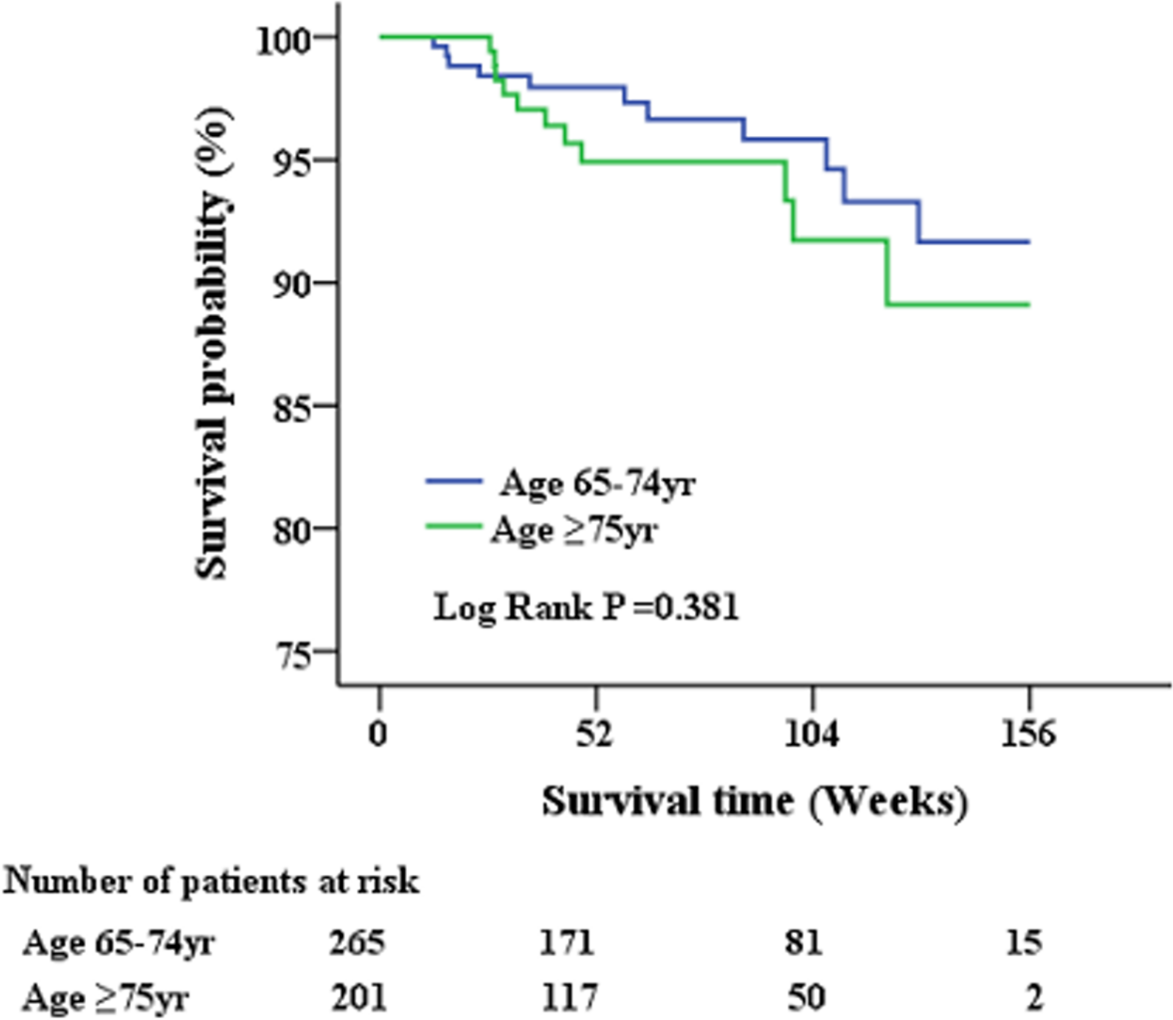
Patient survival after administration of anti-viral therapy. There is no significant survival differences had been observed between early and late elderly patients during the short-term follow-up period.

A total of 479 geriatric patients who completed the treatment course were further analyzed for post-treatment survival (Table 4 and Fig. 2). The index date of the stratified analysis for overall survival was defined by the end-of-treatment with DAA. Baseline factors affecting patient survival after antiviral therapy were analyzed. Accordingly, a higher proportion of patients who succumbed to post-treatment mortality had cirrhosis, history of cancer, ascites, high FIB-4 scores, low albumin levels, low hemoglobin levels, and poor renal function. No differences in HCV genotype, HCV viral load, or type of antiviral agents were observed between survivors and non-survivors. Further multivariate analysis (Table 5) identified cirrhosis (HR: 4.329; 95% CI [1.252–14.973]), albumin level (HR: 0.173; 95% CI [0.065–0.466]) and creatinine level (HR: 1.205; 95% CI [1.054–1.378]) as factors independently associated with post-treatment mortality.

**Table 4:** Comparison of characteristics between survivors and non-survivors after anti-viral therapy.

	**All patients (*N* = 479)**	**Survivors (*N* = 457)**	**Non-survivors (*N* = 22)**	**P value**
Age ≥ 75	208 / 479 (43.4)	197 / 457 (43.1)	11 / 22 (50)	0.677
Male	209 / 479 (43.6)	198 / 457 (43.3)	11 / 22 (50)	0.692
SVR(+)	476 / 479 (99.4)	454 / 457 (99.3)	22 / 22 (100)	1.000
Cirrhosis	217 / 479 (45.3)	199 / 457 (43.5)	18 / 22 (81.8)	0.001
Cancer	70 / 479 (14.6)	62 / 457 (13.6)	8 / 22 (36.4)	0.008
Diabetes mellitus	89 / 479 (18.6)	85 / 457 (18.6)	4 / 22 (18.2)	1.000
Hypertension	146 / 479 (30.5)	140 / 457 (30.6)	6 / 22 (27.3)	0.922
Child–Pugh Classification for cirrhotic patients, n /N(%)				0.506
A	209/217 (96.3)	192/199 (96.5)	17/18 (94.4)	
B	8/217 (3.7)	7/177 (3.5)	1/18(5.6)	
Ascites				0.009
None	463 / 479 (96.7)	444 / 457 (97.2)	19 / 22 (86.4)	0.032
Mild	14 / 479 (2.9)	11 / 457 (2.4)	3 / 22 (13.6)	0.022
Moderate	2 / 479 (0.4)	2 / 457 (0.4)	0 / 22	1.000
FIB4	3.89 (2.64–6.03)	3.87 (2.64–5.91)	6 (3.44–8.86)	0.019
Hepatitis B	26 / 479 (5.4)	24 / 457 (5.3)	2 / 22 (9.1)	0.338
HCV RNA, IU/mL, log10	5.78 (4.81–6.28)	5.8 (4.82–6.29)	5.68 (4.34–6.09)	0.244
CHD	1 / 479 (0.2)	1 / 457 (0.2)	0 / 220	1.000
HIV	6 / 479 (1.3)	5 / 457 (1.1)	1 / 22 (4.5)	0.247
CVA	13 / 479 (2.7)	13 / 457 (2.8)	0 / 220	1.000
ESRD	14 / 479 (2.9)	14 / 457 (3.1)	0 / 220	1.000
BMI	23.57 (21.31–26.06)	23.57 (21.33–26.07)	23.44 (20.7–25.81)	0.71
CRE	0.9 (0.68–1.16)	0.89 (0.68–1.14)	1.04 (0.93–1.75)	0.011
eGFR	73.53 (54.94–90.11)	73.98 (55.67–90.34)	56.62 (38.4–76.08)	0.025
ALT	48 (29–83)	48 (29–83)	48.5 (30–80)	0.795
INR	0.97 (0.92–1.03)	0.97 (0.92–1.03)	0.97 (0.94–1.06)	0.665
BIL	0.74 (0.58–1.01)	0.74 (0.58–1)	0.89 (0.46–1.19)	0.552
ALB	3.8 (3.5–4.1)	3.9 (3.6–4.1)	3.4 (3.2–3.6)	<0.001
HB	13 (11.5–14.2)	13 (11.6–14.3)	12 (10.3–13.5)	0.021

**Notes.**

BMI body mass index ALT alanine aminotransferase BIL bilirubin ALB albumin CRE Creatinine INR international normalized ratio HB haemoglobin FIB-4 Fibrosis-4 CHD coronary heart disease HIV Human immunodeficiency virus CVA cerebrovascular accident HCV hepatitis C virus eGFR estimated Glomerular filtration rate SVR sustained virologic response

**Table 5:** Associations of factors and post treatment survival in univariable and multivariable cox regression analyses.

**Risk Factor**	**Univariate**		**Multivariate**	
	Hazard ratio (95% CI)	*P* value	Hazard ratio (95% CI)	*P* value
Age >75	1.451 (0.628–3.349)	0.384	1.216 (0.511–2.897)	0.659
Male	1.345 (0.583–3.104)	0.487	1.931 (0.767–4.864)	0.163
Cirrhosis	4.009 (1.347–11.93)	0.013	4.329 (1.252–14.973)	0.021
Cancer	2.684 (1.122–6.424)	0.027	2.368 (0.938–5.982 )	0.068
DM	0.867 (0.293–2.566)	0.797	1.131 (0.303–4.222)	0.855
HTN	0.729 (0.285–1.866)	0.51	0.679 (0.225–2.044 )	0.491
ASCITES	5.383 (1.589–18.236)	0.007	1.911 (0.457–7.997)	0.375
FIB4,	1.048 (0.996–1.103)	0.072	–	–
HCV RNA, log10 IU/mL	0.767 (0.532–1.105)	0.154	–	–
Comorbidity number*^a^	1.691 (0.53–5.389)	0.375	–	–
BMI, kg/m^2^	0.986 (0.887–1.097)	0.800	–	–
CRE, mg/dL	1.169 (1.045–1.308)	0.006	1.205 (1.054–1.378)	0.006
eGFR, mL/min/1.73m^2^	0.984 (0.97–0.998)	0.021	–	–
ALT, U/L	0.997 (0.988–1.007)	0.586	–	–
INR	1.056 (0.455–2.451)	0.899	–	–
BIL, mg/dL	1.143 (0.845–1.548)	0.386	–	–
ALB, g/dL	0.167 (0.079–0.356)	<0.001	0.173 (0.065–0.466)	0.001
HB, g/dL	0.775 (0.642–0.936)	0.008	–	–

**Notes.**

BMI body mass index ALT alanine aminotransferase BIL bilirubin ALB albumin CRE Creatinine INR international normalized ratio HB haemoglobin FIB-4 Fibrosis-4 DM diabetes mellitus HTN hypertension HCV hepatitis C virus eGFR estimated Glomerular filtration rate

a Comorbidity number*: CHB, CHD, HIV, and CVA, excluding Cirrhosis, CANCER, DM, and HTN

## Discussion

The current large single-center cohort study on HCV-infected geriatric patients reported an overall SVR rate of 96.7%. Despite the increased prevalence of comorbid conditions and cirrhosis in this population, the high SVR rate obtained herein was comparable with those presented in non-geriatric HCV populations receiving DAA therapy (Chiu et al., 2020; Huang et al., 2020b; Huynh & Hu, 2020; Villani et al., 2019; Wang et al., 2019). Hence, late elderly patients can achieve similarly high SVR rates as early elderly patients with a similar side effect profile. Moreover, the current study identified cirrhosis, low albumin levels, and high creatinine levels as indicators of poor prognosis among the geriatric population after completing DAA therapy in real-world settings.

Compared with the younger population, geriatric patients have complex medical comorbidities that limit responses to HCV treatment in the era of IFN-based therapy (Wu, Pwu & Chen, 2018a; Wu et al., 2018b). Although HCV treatment may reduce not only liver disease progression but also the risk for cardiovascular disease and delay renal dysfunction (Lee et al., 2019; Polo & Laufer, 2017; Soriano et al., 2016; Wu et al., 2018b), IFN-based therapy has not been routinely recommended for the geriatric population (Cainelli, 2008) considering their low response and high withdraw rates (Hu et al., 2013; Roeder et al., 2014). With the introduction of IFN-free therapy for HCV, populations previous considered difficult to treat, such as those with renal dysfunction (Chen et al., 2019; Yen et al., 2020), HIV co-infections (Liu et al., 2020b), or older age (Villani et al., 2019), are currently no longer considered as such.

Elderly subjects are less likely to be enrolled in clinical trials for drug development owing to underlying comorbidities. In fact, Saab et al. (2016), who reviewed four clinical trials for ledipasvir/sofosbuvir with 2,293 patients, revealed that only 12% of patients were ≥65 years of age, whereas only 24 patients were aged ≥75 years. Meanwhile, Foster et al. (2019), who reviewed 9 phase 2 and 3 trials for glecaprevir/pibrentasvir with 2,369 patients, identified only 328 patients (14%) aged ≥65 years. Both regimens have been suggested to be efficacious and safe for the geriatric population. A recent meta-analysis by Villani et al. (2019) found that older and adult patients had similar SVR rates. Accordingly, observational studies showed lower SVR rates than clinical trials (90.1% vs. 96.9%). The current study, which included a large number of late elderly patients aged ≥75 years with a higher proportion of those with liver cirrhosis, revealed findings comparable with those in clinical trials, suggesting the safety of the currently available DAA-based therapy for this population. The low therapy discontinuation rate, high safety profile, and high SVR rate of anti-HCV therapy suggests that age should not be considered a contraindication, unlike IFN-based therapy (Cainelli, 2008).

Current data for DAA therapy in the late elderly population (≥75 years) have been limited. The present study further divided the geriatric population into early and late elderly (Hori et al., 2014). Previous studies had found high adverse event rates in the late elderly population and similar SVR rates among early and late elderly population (Ciaccio et al., 2017; Huynh & Hu, 2020; Villani et al., 2019). Similarly, the present study showed that both patient groups achieved similar high SVR rates. Moreover, both groups showed similar adverse events, except that more early elderly patients had pruritus and anemia than late elderly patients. Pruritus is a common skin disorder in the geriatric population with increased prevalence with age. It is unclear the cause of higher proportion of pruritus reported in our early elderly patient (Cohen et al., 2012). DAA regimen selection at our institution required discussions between the clinician and patient. Considering that current pan-genotypic DAAs offer similar high cure rates and safety profiles, understanding patient preference helps design future therapies that may further enhance patients’ adherence and improve clinical outcomes (Welzel et al., 2019). Interestingly, the present study found that the late elderly population preferred a daily regimen comprising one tablet, suggesting the need for decreasing daily medication burden in this particular population who are at a higher risk of dysphagia (Baijens et al., 2016).

Although most clinical trials and real-world studies have focused more on HCV treatment-related issues (Villani et al., 2019), the present study has been the first to analyze factors associated with short-term post-treatment survival in the geriatric population after having been cured of HCV infection (Table 5). Previous studies have presented considerable evidence confirming that HCV eradication can decrease liver-related and all-cause mortality in adults during a follow-up of 5–10 years (Nuno Solinis et al., 2016). Considering that Taiwanese individuals had a life expectancy of 80.7 years in 2018, understanding post-treatment outcomes especially in the geriatric population is urgently needed for better allocation of medical resources (Ciaccio et al., 2017). Among the 479 patients who completed anti-HCV therapy, 22 (4.6%) succumbed to mortality, with respiratory failure and cardiovascular events being the primary cause of mortality. No significant survival differences had been observed between early and late elderly patients. Moreover, only cirrhosis, albumin level, and creatinine level had been identified as factors independently associated with post-treatment mortality (Table 5). More studies confirming our observations are needed to select better candidates for anti-HCV therapy and improve cost-effectiveness in the geriatric population.

Several limitations of the current study are noteworthy. First, this was a retrospective study with a limited follow-up duration of 3 years, which may be insufficient to demonstrate the benefits of antiviral therapy for the reduction of hepatoma or cardiovascular diseases. Thus, we were unable to further analyze the long-term impact of anti-HCV therapy in the geriatric population. Second, given that 97% of our patients were DAA treatment-naïve with either genotype 1 or 2, our treatment results cannot extrapolate to patients with different genotypes. Considering the retrospective nature of this study, we were unable to compare differences between individual DAAs. Third, considering our inclusion of patients from a single tertiary center who were more likely to have better family support and drug adherence than similarly aged patients, our result cannot be extrapolated to patients from community hospitals or clinics.

## Conclusions

The current real-world study demonstrated that DAA-based therapy was highly effective for geriatric patients.

##  Supplemental Information

10.7717/peerj.10944/supp-1 Data S1 Dataset for analysis

## Additional Information and Declarations

### Data Availability

The following information was supplied regarding data availability: Raw data is available as a Supplemental File.
